# Programmable Topological Magnetic Textures in Above‐Room‐Temperature 2D Ferromagnet Fe_3_GaTe_2_

**DOI:** 10.1002/advs.77160

**Published:** 2026-09-21

**Authors:** Hanqing Shi, Yilian Xi, Jingwei Zhang, Jiaqi Liu, Zhizhen Ren, Shilei Zhang, Weichang Hao, Haifeng Feng, Yi Du

**Affiliations:** ^1^ School of Physics Beihang University Beijing People's Republic of China; ^2^ College of Mathematics and Physics Beijing University of Chemical Technology Beijing People's Republic of China; ^3^ School of Physical Science and Technology ShanghaiTech University Shanghai People's Republic of China

**Keywords:** Fe_3_GaTe_2_, ferromagnet, magnetic skyrmion, skyrmion bag, van der Waals

## Abstract

Topological invariants identify distinct topological states by remaining unchanged under continuous deformations, which reflects the robustness of topological phases against external perturbations. In magnetic systems, they characterize deformation‐resistant spin textures and endow non‐trivial configurations with exceptional stability; however, they also create inherent challenges in precise modulation of topological magnetic states. Here, we demonstrate the generation and programmable modulation of arbitrary topological numbers in a magnetic skyrmion bag system that is thermally stable in the 2D van der Waals (vdW) ferromagnet Fe_3_GaTe_2_ (FGaT) at above room temperature. The surface‐interior energy‐landscape decoupling has been uncovered by exploiting unusual perpendicular magnetic anisotropy (PMA) and the thickness‐dependent demagnetization energy, which enables hierarchical reconstruction of surface branching domains while retaining the bulk topological backbone. The encoding of skyrmion bags with different topological structures is demonstrated to enable applications in binary and higher‐radix information storage. This work establishes a comprehensive strategy for generating, reconfiguring and operating high‐order topological states, which paves the way toward programmable, multi‐state spintronic technologies.

## Graphical abstract

This work demonstrates the programmable generation and deterministic modulation of magnetic skyrmion bags with high‐order topological numbers in the above‐room‐temperature 2D ferromagnet Fe_3_GaTe_2_ (FGaT) via current‐pulse control. Furthermore, highly efficient electrical manipulation and current‐driven parallel motion of these high‐order topological spin textures are realized, paving the way for advanced multi‐state spintronic applications.

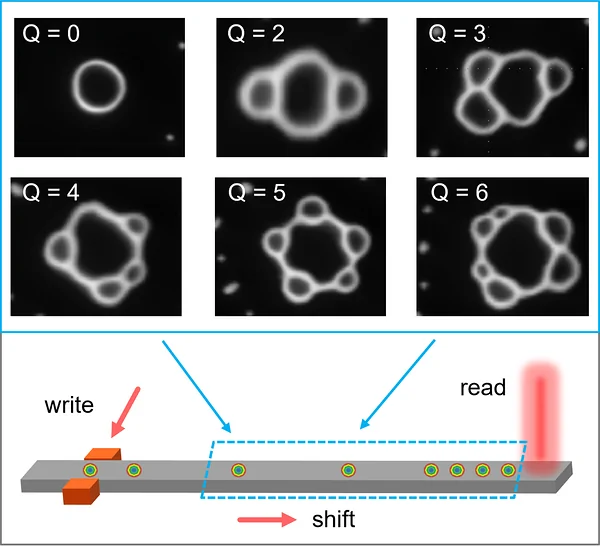

## 1 Introduction

The topological number is an invariant index used to classify distinct topological configurations [1, 2]. In condensed‐matter physics, such invariants underpin a classification framework that goes beyond the traditional Landau paradigm in which phases cannot be distinguished by order parameters. In magnetic textures, it corresponds to the winding number of the normalized local magnetization, providing a quantitative measure of their topological structure [3, 4]. Magnetic domains with non‐trivial topological numbers exhibit remarkable inherent stability due to their topological protection, which means that the external fluctuations of various variables cannot continuously twist the spin into a configuration with different topological numbers [5, 6, 7]. This property endows topologically protected magnetic domains with diverse potential applications in non‐volatile magnetic memory technologies, such as racetrack memory and magnetic random‐access memory (MRAM) [8, 9, 10, 11]. However, while topological protection enhances the stability of magnetic topological textures, it also brings challenges in precise manipulation of the topological number of magnetic domains, especially for increasing the skyrmion information density with varying topological numbers in a single storage unit [3, 6].

Non‐trivial topological domains open a topological protection gap in the energy landscape. Thus, the key to modulating the topological numbers of different spin textures is precisely overcoming the transition energy barriers [4, 12, 13]. Generally, modulating methods such as field cooling and current pulses provide sufficient energy to overcome the energy barrier for the generation of skyrmions [14, 15, 16, 17]. However, considering the 3D configurations of skyrmions, known as skyrmion tubes, where the magnetic moments stack uniformly along the tubes, the thermal activation induced by field cooling and current pulses can indiscriminately disrupt the magnetic order across the entire system [18, 19]. Therefore, such approaches only enable unidirectional transitions from topologically trivial states to skyrmions or skyrmion bags, but fail to achieve multi‐level modulation of the topological number, especially the transition from low‐order to high‐order skyrmion bags. A key requirement for industrial applications is the development of skyrmion bags that remain stable at room temperature under a small magnetic field, coupled with the ability to convert between bags of different topological numbers. Interestingly, if the material has sufficiently strong perpendicular magnetic anisotropy, the enhancement of demagnetizing energy at the open boundary of a bulk magnet will alter the local energy density configuration, subsequently modifying the surface magnetic structure [20, 21]. It is expected that skyrmion bags with arbitrary topological numbers can be realized in this class of materials by precisely controlling the external energy input, so that the surface energy overcomes the topological barrier while keeping the interior energy below this threshold. The recently emerged 2D vdW ferromagnetic Fe_3_GaTe_2_ has strong PMA. Its demagnetization energy strongly depends on thickness [22, 23, 24]. More importantly, Fe_3_GaTe_2_ exhibits a record‐high Curie temperature (*T*_C_) of 350–380 K, which is critical to develop industrial implementation in spintronics.

Here, by engineering the excitation and relaxation pathways through coordinated magnetic‐field and current‐pulse control in the above‐room‐temperature 2D magnet Fe_3_GaTe_2_, we achieve reversible and deterministic transitions between skyrmion bags carrying different topological numbers. This capability further allows topological magnetic textures to serve as robust information units, thereby enabling binary and higher‐radix encoding schemes within a single reconfigurable magnetic texture. These results establish a general route for controllably reshaping topological spin structures in vdW magnets, and set the stage for multi‐state, high‐density spintronic architectures based on programmable topological degrees of freedom.

## 2 Magnetic Properties of FGaT Crystals

**FIGURE 1:**
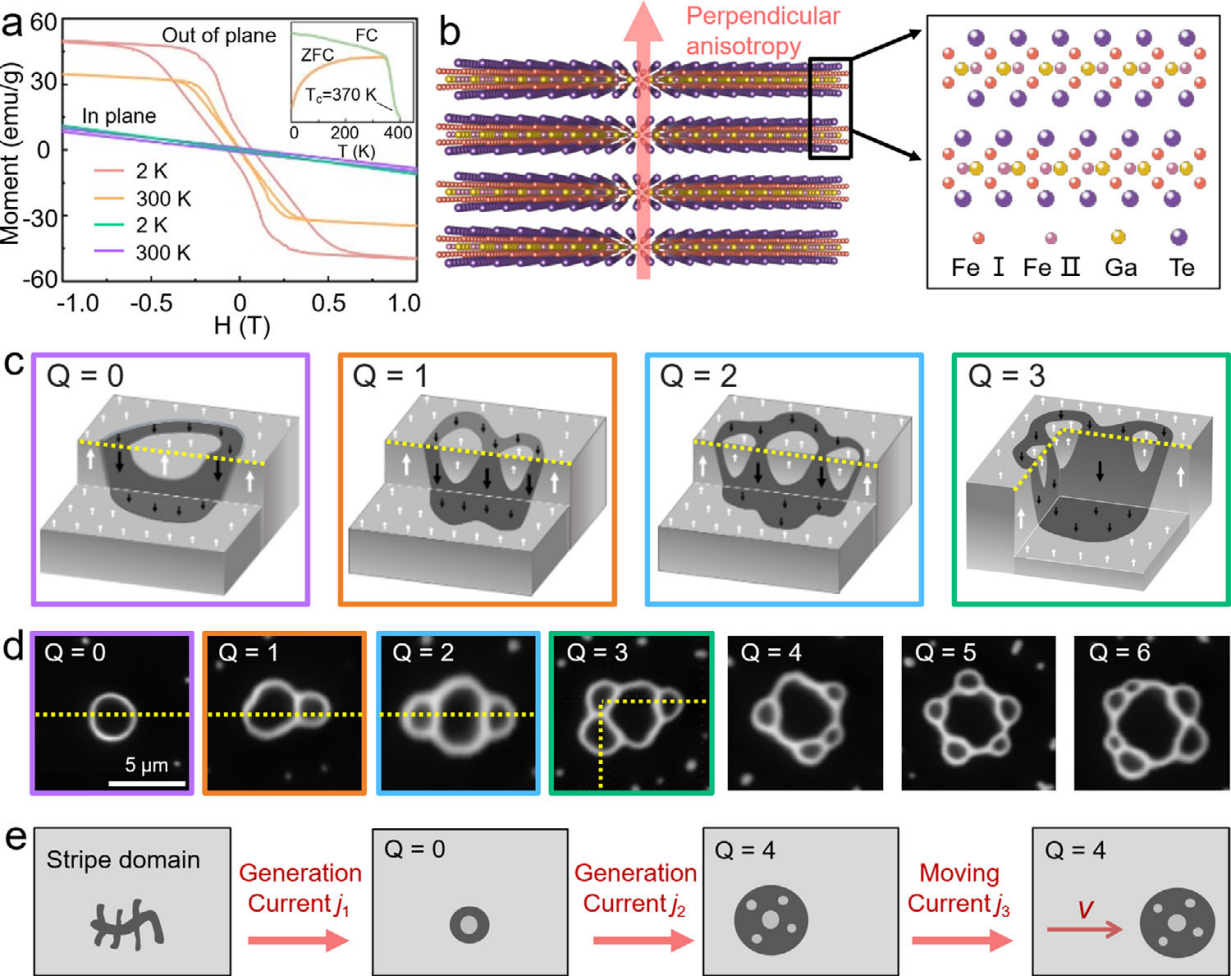
Magnetic properties of FGaT crystals. (a) Magnetization of FGaT measured with the magnetic field perpendicular and parallel to the *c* axis at various temperatures. The inset shows the FC and ZFC curves of FGaT with an external field of 100 mT. (b) Perpendicular anisotropy of FGaT and single‐crystal structure of FGaT along *c* axis. (c) The definition of magnetic skyrmion bags with different Q numbers. (d) MFM images of skyrmion bags with Q values from 0 to 6. (e) Schematics of generation, regulation, and motion of skyrmion bags under current pulses.

FGaT, as a rare above‐room‐temperature 2D ferromagnetic material, exhibits a typical 2D vdW layered structure, where the Fe_3_Ga slab is sandwiched by Te layers (Figure 1b). The synthesized FGaT crystals exhibit a *T*_C_ of 370 K, as illustrated in Figure 1a. This record‐breaking *T*_C_ is mainly caused by the shift of the localized Fe band toward the Fermi level as well as the enhanced magnetic exchange effect by the strong itinerant ability of Fe atoms [24]. The out‐of‐plane and in‐plane magnetization hysteresis loops reveal strong PMA in FGaT single crystals. It is known that the strong PMA can suppress thermal fluctuations, thereby stabilizing the magnetic order in 2D magnetic materials [25]. In magnetic materials with strong PMA, magnetic domains are attributed to the competition between domain wall energy and demagnetization energies [26]. As a typical intrinsic 2D ferromagnetic material with strong PMA, the magneto‐optical Kerr effect (MOKE) microscopy images of FGaT with a thickness of 3 µm show labyrinthine stripe domains (Figure S1). We also conducted magnetic force microscopy (MFM) measurement in FGaT sample with a thickness of 25 µm (Figure S2). We found a distinct domain structure in this sample, where circular skyrmion domains are embedded in stripe domains (Figure S3). According to the classic two‐phase branching model [27] (Figure S4), thick magnetic samples can develop branched surface domains due to the competition between domain‐wall and magnetostatic energies (Note S1). Once the sample thickness exceeds a critical value, surface domain branching occurs (Figure S5). Experimentally, the critical thickness is determined to be approximately 10 µm. Meanwhile, an important prediction of this model is that the width of the surface‐branching domains does not increase indefinitely with sample thickness, but instead approaches a stable value (*W_S_*), which we experimentally measured to be $W_{S}\approx 0.5\;\mu m∼2\;\mu m$ (Figure S6). Specifically, narrowed surface domains suppress stray fields, while wider interior domains minimize the total domain wall length [20, 21]. We systematically investigated FGaT samples with thicknesses ranging from 0.1 to 10 µm and observed stable skyrmions (Figures S5 and S7). Lorentz transmission electron microscope (LTEM) measurements directly confirmed that the magnetic textures are Néel‐type skyrmions (Figure S8). Previous studies, including our own work, have established the presence of DMI in FGaT [22, 28, 29], which pointed out that the value of DMI is 1.048 mJ/m^2^. Such a DMI strength is sufficient to substantially lower the energy of chiral domain walls and promote skyrmion bag formation. Therefore, when DMI is included [30], the energy of chiral domain walls is reduced according to $\gamma_{w}=4\sqrt{AK_{u}}-\pi D$. As a consequence, magnetic configurations containing closed chiral domain‐wall loops become energetically favorable. Consequently, the branched stripe domains generated near the sample surface evolve toward skyrmion‐bag configurations during the FC process.

We further performed MFM measurements and an exfoliation experiment to identify the surface‐branching domain. The lift‐height‐dependent MFM signals indicate that the weak‐stray‐field regions are strongly localized (Figures S9 and S10), consistent with surface‐confined domain branching, whereas the stronger stray fields originate from bulk domains. Importantly, after mechanically removing ∼5 µm of the surface layer from a 20 µm‐thick sample, all skyrmion bags transformed into conventional skyrmions in the same regions (Figure S11), providing direct evidence that the skyrmion bags are confined to the surface region.

The branched domains inherently form complex topological structures. We introduce the Q number as a topological number to label distinct skyrmion bag morphologies (Figure 1c). For example, skyrmion bubbles display a Q value of −1, while skyrmionium, a toroidally structured closed‐loop domain, exhibits a topological charge of Q = 0. A skyrmion bag containing two nested closed loops manifests a Q value of 1. Apart from the skyrmion bags with fixed Q values, we fabricated skyrmion bags with varying Q values in the FGaT system (Figure 1d). Typically, such skyrmion bag systems exhibit a hierarchical architecture comprising a predominant magnetic domain as the central core, circumscribed by an array of smaller subdomains. The structurally intricate skyrmion bag domains are stabilized by the strong magnetic anisotropy of FGaT, suppressing the collapse of skyrmion bags caused by thermal disturbances [31]. The generated skyrmion bags can remain stable in air for one year (Figure S12). Additionally, their information‐encoding capacity (such as varying Q values) endows them with the potential to serve as versatile platforms for next‐generation nonvolatile storage technologies. As shown in Figure 1e, a current‐based modulation technique is developed, which facilitates the transformation from stripe domains to skyrmion bags with arbitrary Q values through current injection. Current injection can manipulate the motion of these skyrmion bags with different Q values. These advances highlight the potential of FGaT for implementing reconfigurable spintronic architectures and racetrack‐type memory systems [32].

### 2.1 Generation of Skyrmion Bags with Arbitrary Topological Numbers

**FIGURE 2:**
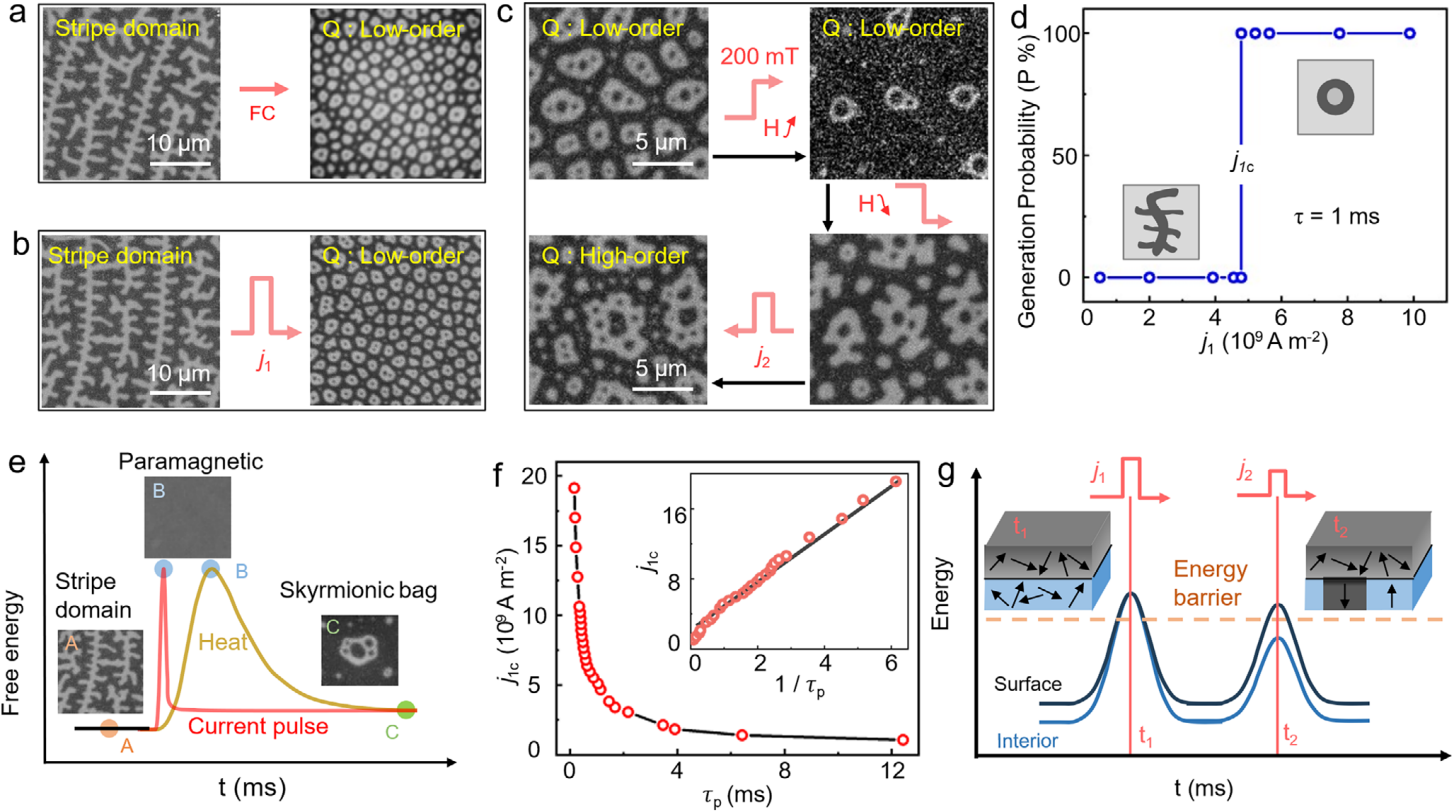
Electromagnetically driven generation of magnetic skyrmion bag states. (a) Formation of skyrmion bags via field‐cooling process. (b) Generation of skyrmion bags by current pulse excitation (*j*_1_ = 7.8 ×10^9^ A/m^2^, τ = 0.5 ms, H = 30 mT). (c) Generation process of high‐Q value skyrmion bags by applying a combination of magnetic field and current pulse. (d) Correlation between generation probability and current density. (e) Energy schematic for two different generation methods. (f) Relationship between critical current density and pulse width required for skyrmion bag nucleation. (g) Schematic illustration of the formation mechanism of high‐Q skyrmion bag states.

In the FGaT system, skyrmion bags with arbitrary topological numbers can be generated by field‐cooling and electric current‐driven methodology. Through the field‐cooling method, the labyrinthine domains transform into skyrmions in a 102 nm‐thick FGaT sample (Figure S13). Through the same method, however, skyrmion bags can be generated in 25 µm‐thick samples (Figure 2a). These skyrmion bags exhibit small Q values, ranging from ‐1 to 1. Notably, skyrmion bags with similar Q values can be generated by current pulse *j*_1_, as illustrated in Figure 2b. Compared with conventional thermal activation, current pulses can reach a millisecond timescale, which is six orders of magnitude shorter than that of heating. To realize stable, arbitrarily high‐order skyrmion bags, we use lower subthreshold current density pulses *j*_2_ (*j*_2_< *j*_1_) to induce hierarchical amplification of Q values from low‐order bags, as shown in Figure 2c. The protocol proceeds through two manipulation processes using a magnetic field pulse and a current pulse. First, the magnetic field pulse annihilates metastable domains and then permits spatial reconfiguration of surviving domains. Second, subthreshold current density pulse *j*_2_ induces non‐destructive restructuring, where retained bags acquire higher‐order topology through controlled spin torque injection. Figure 2d shows the current‐driven evolution of the magnetic domain. Once the current density exceeds the critical threshold (*j* > *j*_c_), the transition from stripe domains to skyrmion bags occurs, indicating an intrinsic coherent mechanism governing the transformation. The current injection induces a thermal effect, known as Joule heating, which raises local temperatures to the Curie temperature (T > *T_C_*). This reveals a mechanistic consistency between electrical and thermal activation pathways, where both methods break ferromagnetic order through thermal fluctuations, thereby enabling field‐induced reconfiguration into skyrmion bags during the subsequent FC process (Figure 2e). By integrating Zeeman energy from applied magnetic fields and accounting for demagnetization effects, the skyrmion bag configuration emerges as the thermodynamic ground state (Figure S14). This transition mechanism is further validated by the inverse proportionality between the *j_1c_* and pulse duration (*τ*), as plotted in Figure 2f. This inverse relationship is attributed to the arising temperature induced by Joule heating, which reduces the pinning barrier of magnetic domain walls [33, 34]. However, although this process is driven by Joule heating of the current, it remains controllable. By precisely tuning the current density and pulse duration, the magnetic texture can be controllably transformed from lower‐order to higher‐order skyrmion‐bag states, resulting in a stepwise increase of the topological index (Figure S15). To better understand the formation mechanism of these skyrmion bags, the corresponding energy landscape is illustrated in Figure 2g. The subthreshold current‐density pulses (*j*_2_) induce a transition from low‐order to high‐order skyrmion bags without triggering a complete reconstruction of the magnetic domains. This observation suggests that the evolution proceeds through a hierarchical transformation inherited from the pre‐existing magnetic configuration. We attribute this behavior to a surface‐bulk energetic differentiation established by surface domain branching. As discussed in Note S1, the branched surface domains possess a higher free‐energy density than the interior domains, resulting in a preferential response of the surface magnetic texture under *j*_2_ excitation while preserving the skyrmion configuration in the bulk. Consequently, the bulk magnetic structure serves as a magnetic template that guides the reconstruction of the surface domains, ultimately leading to the formation of higher‐order skyrmion bags.

### 2.2 Coding Based on the Modulation of Topological Charge of Skyrmion Bags States

**FIGURE 3:**
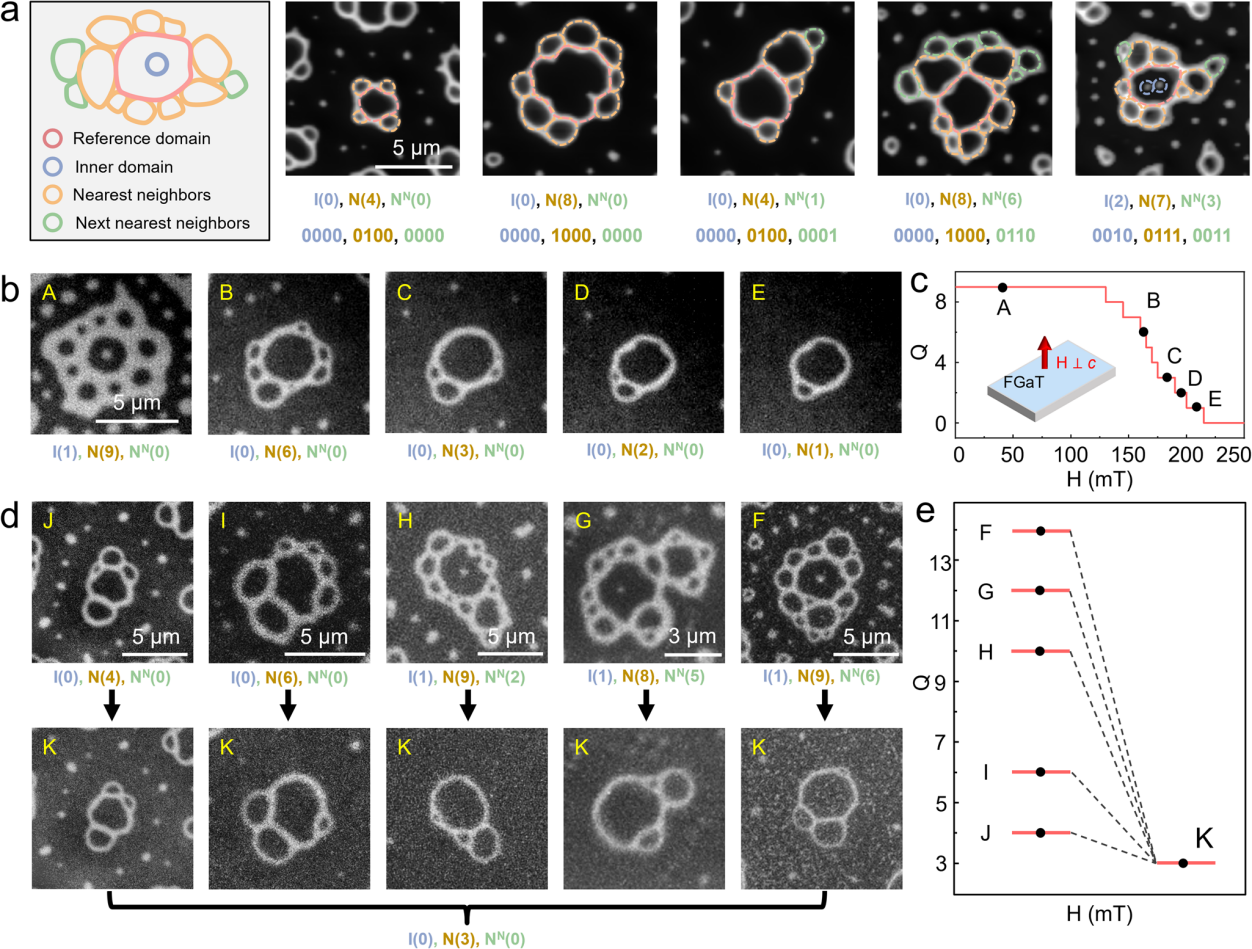
Coding based on the modulation of the topological number of skyrmion bag states. (a) The coding rules adopt the numbers of three different magnetic domains: inner domain (I), nearest neighbors (N), next‐nearest neighbors (N^N^). (b‐c) The skyrmion bag with an initial state Q = 9 undergoes a transformation of its Q value to a lower order as the magnetic field increases. (d‐e) Skyrmion bags with different initial states are transformed into the same type of skyrmion bags under a magnetic field.

Skyrmion bags with distinct Q values manifest distinct domain structures. They are characterized by maximal spatial extent, internal subdomains potentially harboring negative Q values, and numerous smaller circular domains radially organized around the largest one, forming distinct nearest‐neighbor and next‐nearest‐neighbor spatial arrangements. Thus, a coding scheme can be defined according to the number of three distinct magnetic domain types. As shown in Figure 3a, the number of inner domains (I), nearest‐neighbor domains (N), and next‐nearest‐neighbor domains (N^N^) can be used to encode. For instance, a domain configuration denoted as [I (2), N (7), N^N^ (3)] could encode the binary sequence (0010, 0111, 0011). This encoding scheme is analogous to previous skyrmion bag‐based encoding approaches. In this way, 12‐bit binary coding can be realized. Furthermore, the topological states of the skyrmion bags can be further manipulated by tuning the magnitude of the external magnetic field. As demonstrated in Figure 3b,c, the initial Q value of the skyrmion bag (I (1), N (9), N^N^ (0)) reduces step by step from 9 to 1 (A—E) with increasing magnetic field. The annihilation process follows a hierarchical degradation pathway. It begins with the preferential disruption of the internal domains within the primary magnetic structure, followed by the progressive shrinkage and ultimately disappearance of the adjacent domains. As the magnetic field continues to increase, these domains transform into circular domains with a Q value of 0, ultimately collapsing into a ferromagnetic state as the field approaches saturation. In addition, the high‐order skyrmion bags with different Q values can be modulated by external fields, converging into the low‐order skyrmion bags with identical Q values. As shown in Figure 3d,e, five distinct skyrmion bags with Q values ranging from 6 to 14 can transform into a common state with Q = 3 under an applied magnetic field. In summary, while current pulses tend to increase the Q value of skyrmion bags, external magnetic fields tend to decrease it, thereby resetting the magnetic state of the device. The combined use of these two approaches can thereby be integrated for complete all‐electromagnetic generation and manipulation of skyrmion bags.

Compared with conventional skyrmion‐based memory devices, which typically encode binary information based on the presence or absence of individual skyrmions (0/1 states), higher‐order topological states provide additional internal degrees of freedom, including distinct topological numbers and configurational variations. As a result, a single higher‐order structure can encode multiple bits of information (12 bits in our implementation), leading to significantly enhanced information storage density.

### 2.3 Current Drive Dynamics of Skyrmion Bags

**FIGURE 4:**
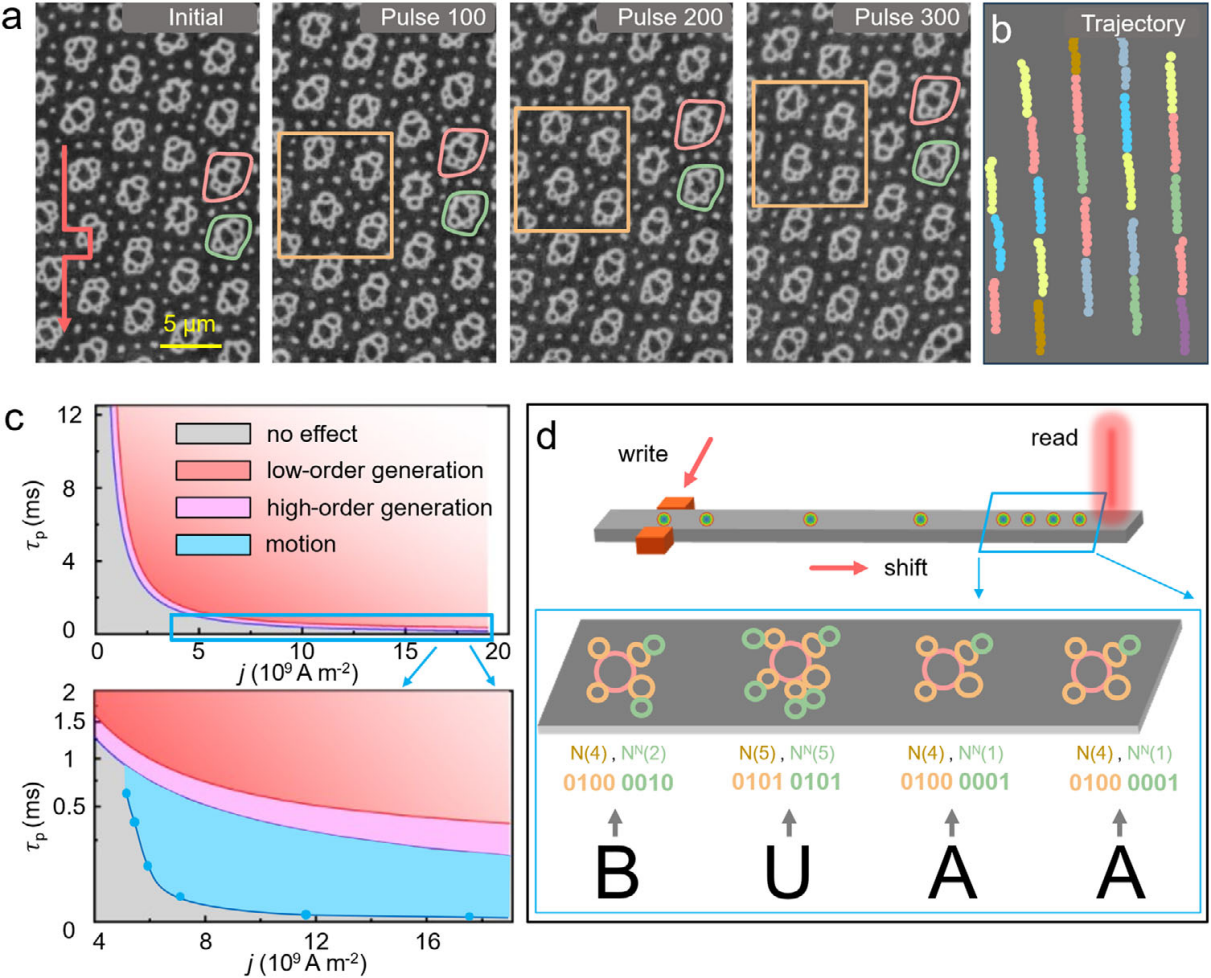
Current drive characteristics of skyrmion bags. (a) MOKE images of skyrmion bags acquired before the current pulse and after every 100 current pulses along the + y direction, with current density of *j*_3_ = 8 × 10^9^ A/m^2^ and a pulse width of 10 µs. The highlighted regions in orange, pink, and green are typical skyrmion bags. (b) The trajectory of each magnetic skyrmion bag. (c) The current density versus pulse width phase diagram. (d) A schematic image of a possible integrated device for full electromagnetic generation, regulation, driving, and readout of magnetic skyrmion bag states.

The current‐driven mobility of magnetic domains serves as a critical prerequisite for their practical implementation in racetrack memory systems [35, 36]. The current‐driven dynamics of skyrmion bags in FGaT are systematically investigated. We used a liquid‐metal (GaInSn) contacted current‐injection device (Figure S16) to achieve highly efficient electrical coupling with the FGaT crystal, ensuring homogeneous current injection and uniform current‐density distribution. Figure 4a shows the dynamic responses of skyrmion bags with high‐Q values by time‐resolved MOKE tracking, where skyrmion bags without inner domains (I (0)) move along the + *y*‐axis under continuous current pulses (*j*_3_ = 8 × 10^9^ A/m^2^, τ = 10 µs). The thermal effects induced by such short‐pulse currents are insufficient to disrupt the structure of skyrmion bags or nucleate additional skyrmion bags. As shown in Figure 4b, the skyrmion bags undergo a total displacement of 6.03 µm after 300 pulses, from which the speed of the skyrmion bags is calculated to be 2.01 mm/s. Besides, the higher‐order skyrmion bags (N^N^ > 0) and lower‐order skyrmion bags (Q = 0), can also be propelled by current (Figures S17 and S18). Unlike the mechanism by which current generates skyrmion bags via thermal effects, this current‐induced movement is attributed to the spin transfer torque (STT) effect of the spin‐polarized current, which is generated by the spin‐filtering of the out‐of‐plane magnetic moments of FGaT [37, 38]. The STT‐driven motion is further proved by the observation that the skyrmion bags move parallel to the electron flow (Videos S1 and S2). When a higher current density is applied at a fixed pulse width, their velocity increases significantly (Figure S19), demonstrating a positive correlation between the current density and the motion velocity of skyrmion bags.

It can be observed that the trajectory of the skyrmion bag is parallel to the current direction. We attribute this to the following main reasons. When encountering locally distorted edge regions, the skyrmion bag circumvents them and returns to its original trajectory (Video S3), indicating a repulsive interaction associated with the sample boundary. According to previous theoretical and micromagnetic studies [39], the magnetization twists near the edge can generate an edge‐induced repulsive potential, which prevents the magnetic texture from being annihilated at the sample boundary. Moreover, the dynamics are further influenced by collective interactions among neighboring skyrmion bags. The skyrmion bags exhibit intrinsic bag‐bag repulsive interactions [40], which suppress lateral aggregation. In addition, a high‐density ensemble of interstitial skyrmions residing between neighboring skyrmion bags enables relative sliding (Video S4 and Figure S20), effectively acting as a “lubricating” medium. These lubricating skyrmions can balance the inter‐bag interactions to ensure the collective motion of the skyrmion bags and prevent mutual “wear” that could lead to loss of information. Therefore, the edge confinement, skyrmion‐bag interactions, and bag‐bag repulsive interactions suppress the transverse displacement expected for an isolated skyrmion, resulting in the nearly current‐parallel motion observed experimentally.

Our experimental results establish the current pulse as a versatile and effective tool for the generation, modulation, and motion of skyrmion bags. As illustrated in the current density‐pulse width (*J*‐τ) phase diagram in Figure 4c, a current density satisfying the criterion *j*_c_ · *τ* > *C*_2_ enables the formation of lower‐order skyrmion bags. Marginally reduced currents (e.g., C_1_< *j*_c_·τ < C_2_) can further induce a transition to higher‐order skyrmion bags. Notably, these generated skyrmion bags can then be modulated into lower‐Q states through an external magnetic field. Furthermore, currents with higher densities and narrower pulse widths are capable of driving all types of skyrmion bags without structural disruption. The operating current density is typically on the order of 10^9^ A/m^2^, which is considerably lower than that commonly reported for multilayer skyrmion racetrack memory systems [36]. Consequently, it is feasible to integrate all components into a single device, thereby realizing the comprehensive electromagnetic generation, modulation, propulsion, and readout of skyrmion bags. As illustrated in Figure 4d, current‐density pulses can be applied via transverse electrodes to drive the motion of magnetic domains across the entire track. The longitudinal electrodes further enable precise control. The high‐density current generates low‐order skyrmion bags, while low‐density current promotes the high‐order transformation of these skyrmion bags. Throughout operational cycles, the topological charge (Q) of the bags can be tuned on demand via magnetic field control, enabling real‐time reconfiguration for targeted information encoding. Furthermore, information readout can be achieved through non‐invasive optical interrogation techniques, MFM, or electrical transport methods, including tunnel magnetoresistance (TMR) and topological Hall effect (THE) [10, 41]. Employing the previously described encoding scheme, binary ASCII codes can be used to represent English letters (Table S1). For example, the uppercase letter “A” can be uniquely represented by the binary code 01000001. Within the skyrmion bag system, this code can be split into 0100 and 0001, corresponding to skyrmion bags with four neighbors and one next‐nearest neighbor. Extending this method, letters like “U” and “B” can be similarly encoded, thus forming “BUAA” (the English abbreviation for Beihang University, Beijing, China). The generation and motion of skyrmion bags arise from the competition between the thermal effect and the STT effect generated by current, where STT becomes dominant at smaller current pulse widths, thus driving the skyrmion bags.

## 3 Conclusion

In summary, we systematically unveil the generation mechanisms and dynamic behaviors of skyrmion bags under current control, providing theoretical foundations and technical pathways for constructing high‐efficiency, programmable spintronic devices. By precisely modulating current density and pulse width parameters, not only can the topological states (Q values) of skyrmion bags be controlled, but also their state evolution and information encoding can be synergistically optimized through magnetic field assistance. This multi‐dimensional control capability breaks the static limitations of traditional magnetic domains, opening new avenues for next‐generation low‐power, high‐density information storage and logic units.

## 4 Methods

### 4.1 Sample Preparation

FGaT crystals were grown using the self‐flux method. Highly pure Fe (99.9%), Ga (99.99%), and Te (99.99%) powders, with a mole ratio of 1:1:2, were mixed under an Ar atmosphere in a glove box and sealed in a quartz tube under 1.9 Pa. To capture the excess flux during centrifugation, a crucible filled with quartz wool was placed on top of the growth crucible. The melt was homogenized at 1000°C for approximately 24 h, then cooled quickly to 880°C within 1 h, followed by slowly cooling to 780°C in 100 h. At this temperature, the ampoules were removed from the furnace and placed in a centrifuge to expel the excess flux. Then, large single crystals could be obtained.

### 4.2 Device Fabrication and Current Injection

FGaT crystals were mechanically exfoliated to remove the naturally formed surface oxidation layer and subsequently cut into a strip‐shaped geometry with approximately parallel edges. Electrical contacts were formed using a liquid‐metal GaInSn alloy, which provides excellent electrical coupling with the FGaT crystal while introducing negligible contact resistance. The two liquid‐metal electrodes were arranged in a parallel configuration to ensure a uniform current density distribution across the sample. The current density was calculated as $j=\frac{V}{R}\;\cdot \frac{1}{w\cdot d}$ where V is the applied voltage, R is the device resistance, w is the sample width, and d is the sample thickness, both determined by an optical microscope.

### 4.3 Magnetic Measurements and Imaging

The magnetic properties were measured by a Physical Properties Measurement System (PPMS, Quantum Design with a 9 T magnetic field), MOKE, and magnetic force microscope (MFM). MFM measurements were conducted in Lift Mode in air at room temperature using a Bruker Icon system for conventional imaging and a Quantum Scale AFM‐NanoTech 20 system with a customized sample stage for in situ measurements. The magnitude of the magnetic field was controlled by adjusting the distance between the sample and the magnet. The measurements were performed at room temperature in air.

## Author Contributions

**Hanqing Shi**: conceptualization, writing – original draft, writing – review and editing, visualization, validation, methodology, investigation, funding acquisition, software, formal analysis, project administration, data curation, supervision, resources. **Yilian Xi**: conceptualization, investigation, funding acquisition, methodology, validation, visualization, project administration, writing – review and editing, formal analysis, software, resources, supervision, data curation, writing – original draft. **Jingwei Zhang**: conceptualization, methodology, formal analysis, validation, visualization, investigation, funding acquisition, Writing – review and editing, Writing – original draft, project administration, software, resources, supervision, data curation. **Jiaqi Liu**: methodology. Zhizhen Ren: methodology. **Shilei Zhang**: resources, supervision, project administration, formal analysis, writing – review and editing, visualization, validation, funding acquisition, investigation. **Weichang Hao**: writing – review and editing, visualization, validation, investigation, funding acquisition, project administration, formal analysis, resources, supervision. **Haifeng Feng**: writing – review and editing, visualization, validation, investigation, funding acquisition, project administration, formal analysis, resources, supervision. **Yi Du**: writing – review and editing, validation, visualization, funding acquisition, investigation, project administration, resources, supervision, formal analysis.

## Conflicts of Interest

The authors declare no conflicts of interest.

## Supporting information




**Supporting File 1**: advs77160‐sup‐0001‐SuppMat.docx.

**Supporting File 2**: advs77160‐sup‐0002‐VideoS1.mp4.

**Supporting File 3**: advs77160‐sup‐0003‐VideoS2.mp4.

**Supporting File 4**: advs77160‐sup‐0004‐VideoS3.mp4.

**Supporting File 5**: advs77160‐sup‐0005‐VideoS4.mp4.

## Data Availability

All data are available in the main text or the supplementary materials. Further data are available from the corresponding author on reasonable request.

## References

[1] S.‐W. Wei , Y.‐X. Liu , and R. B. Mann , “Black Hole Solutions as Topological Thermodynamic Defects,” Physical Review Letters 129, no. 19 (2022): 191101, 10.1103/physrevlett.129.191101. 36399744

[2] S. Shankar , A. Souslov , M. J. Bowick , M. C. Marchetti , and V. Vitelli , “Topological Active Matter,” Nature Reviews Physics 4, no. 6 (2022): 380–398, 10.1038/s42254-022-00445-3.

[3] A. N. Bogdanov and C. Panagopoulos , “Physical Foundations and Basic Properties of Magnetic Skyrmions,” Nature Reviews Physics 2, no. 9 (2020): 492–498, 10.1038/s42254-020-0203-7.

[4] N. Nagaosa and Y. Tokura , “Topological Properties and Dynamics of Magnetic Skyrmions,” Nature Nanotechnology 8, no. 12 (2013): 899–911, 10.1038/nnano.2013.243. 24302027

[5] A. Fert , N. Reyren , and V. Cros , “Magnetic Skyrmions: Advances in Physics and Potential Applications,” Nature Reviews Materials 2, no. 7 (2017): 17031, 10.1038/natrevmats.2017.31.

[6] Y. Tokura and N. Kanazawa , “Magnetic Skyrmion Materials,” Chemical Reviews 121, no. 5 (2021): 2857–2897, 10.1021/acs.chemrev.0c00297. 33164494

[7] A. Rana , C.‐T. Liao , E. Iacocca , et al., “Three‐dimensional Topological Magnetic Monopoles and Their Interactions in a Ferromagnetic Meta‐lattice,” Nature Nanotechnology 18, no. 3 (2023): 227–232, 10.1038/s41565-022-01311-0. 36690739

[8] S. Parkin and S.‐H. Yang , “Memory on the Racetrack,” Nature Nanotechnology 10, no. 3 (2015): 195–198, 10.1038/nnano.2015.41. 25740128

[9] S. S. P. Parkin , M. Hayashi , and L. Thomas , “Magnetic Domain‐wall Racetrack Memory,” Science 320, no. 5873 (2008): 190–194, 10.1126/science.1145799. 18403702

[10] S. Chen , J. Lourembam , P. Ho , et al., “All‐electrical Skyrmionic Magnetic Tunnel Junction,” Nature 627, no. 8004 (2024): 522–527, 10.1038/s41586-024-07131-7. 38509277

[11] X. Zhang , M. Ezawa , and Y. Zhou , “Magnetic Skyrmion Logic Gates: Conversion, Duplication and Merging of Skyrmions,” Scientific Reports 5, no. 1 (2015): 9400, 10.1038/srep09400. PMC4371840 25802991

[12] D. Cortés‐Ortuño , W. Wang , M. Beg , et al., “Thermal Stability and Topological Protection of Skyrmions in Nanotracks,” Scientific Reports 7 (2017): 4060, 10.1038/s41598-017-03391-8. PMC5481343 28642570

[13] J. C. Criado , P. D. Hatton , Á. Lanza , S. Schenk , and M. Spannowsky , “Charting the Free Energy Landscape of Metastable Topological Magnetic Objects,” Physical Review B 109, no. 19 (2024): 195114, 10.1103/physrevb.109.195114.

[14] M. T. Birch , L. Powalla , S. Wintz , et al., “History‐Dependent Domain and Skyrmion Formation in 2D Van der Waals Magnet Fe_3_GeTe_2_ ,” Nature Communications 13, no. 1 (2022): 3035, 10.1038/s41467-022-30740-7. PMC9156682 35641499

[15] A. Chakraborty , A. K. Srivastava , A. K. Sharma , et al., “Magnetic Skyrmions in a Thickness Tunable 2D Ferromagnet From a Defect Driven Dzyaloshinskii–Moriya Interaction,” Advanced Materials 34, no. 11 (2022): 2108637, 10.1002/adma.202108637. PMC11475517 35048455

[16] S. Yang , K.‐W. Moon , T.‐S. Ju , et al., “Electrical Generation and Deletion of Magnetic Skyrmion‐Bubbles via Vertical Current Injection,” Advanced Materials 33, no. 45 (2021): 2104406, 10.1002/adma.202104406. PMC11469294 34569658

[17] I. Lemesh , K. Litzius , M. Böttcher , et al., “Current‐Induced Skyrmion Generation Through Morphological Thermal Transitions in Chiral Ferromagnetic Heterostructures,” Advanced Materials 30, no. 49 (2018): 1805461, 10.1002/adma.201805461. 30368960

[18] D. Wolf , S. Schneider , U. K. Rößler , et al., “Unveiling the Three‐dimensional Magnetic Texture of Skyrmion Tubes,” Nature Nanotechnology 17, no. 3 (2022): 250–255, 10.1038/s41565-021-01031-x. PMC8930765 34931032

[19] S. Seki , M. Suzuki , M. Ishibashi , et al., “Direct Visualization of the Three‐dimensional Shape of Skyrmion Strings in a Noncentrosymmetric Magnet,” Nature Materials 21, no. 2 (2022): 181–187, 10.1038/s41563-021-01141-w. 34764432

[20] R. Gemperle , “A Contribution to the Study of the Surface Domain Structure of Uniaxial Ferromagnets,” Physica Status Solidi b 6 (1964): 89, 10.1002/pssb.19640060106.

[21] F. Otto and T. Viehmann , “Domain Branching in Uniaxial Ferromagnets: Asymptotic Behavior of the Energy,” Calculus of Variations and Partial Differential Equations 38, no. 1‐2 (2010): 135–181, 10.1007/s00526-009-0281-y.

[22] H. Shi , J. Zhang , Y. Xi , et al., “Dynamic Behavior of Above‐room‐temperature Robust Skyrmions in 2D van der Waals Magnet,” Nano Letters 24, no. 36 (2024): 11246–11254, 10.1021/acs.nanolett.4c02835. 39207036

[23] G. Zhang , F. Guo , H. Wu , et al., “Above‐room‐temperature Strong Intrinsic Ferromagnetism in 2D van der Waals Fe_3_GaTe_2_ With Large Perpendicular Magnetic Anisotropy,” Nature Communications 13, no. 1 (2022): 5067, 10.1038/s41467-022-32605-5. PMC9424191 36038556

[24] Y. Xi , H. Shi , J. Zhang , et al., “Large Magnetic Anisotropy in van der Waals Ferromagnet Fe_3_GaTe_2_ Above Room Temperature,” The Journal of Physical Chemistry Letters 15, no. 43 (2024): 10802–10810, 10.1021/acs.jpclett.4c02426. 39432822

[25] C. Gong , L. Li , Z. Li , et al., “Discovery of Intrinsic Ferromagnetism in Two‐dimensional van der Waals Crystals,” Nature 546, no. 7657 (2017): 265–269, 10.1038/nature22060. 28445468

[26] Q. Li , M. Yang , C. Gong , et al., “Patterning‐induced Ferromagnetism of Fe_3_GeTe_2_ van der Waals Materials Beyond Room Temperature,” Nano Letters 18, no. 9 (2018): 5974–5980, 10.1021/acs.nanolett.8b02806. 30114354

[27] A. Hubert and R. Schäfer , Magnetic Domains: The Analysis of Magnetic Microstructures (Springer, 1998), 10.1007/978-3-540-85054-0.

[28] Z. Li , H. Zhang , G. Li , et al., “Room‐TEMPERATURE sub‐100 nm Néel‐type Skyrmions in Non‐Stoichiometric Van der Waals Ferromagnet Fe_3‐x_GaTe_2_ With Ultrafast Laser Writability,” Nature Communications 15, no. 1 (2024): 1017, 10.1038/s41467-024-45310-2. PMC10838308 38310096

[29] C. Zhang , Z. Jiang , J. Jiang , et al., “Above‐Room‐Temperature Chiral Skyrmion Lattice and Dzyaloshinskii–Moriya Interaction in a Van der Waals Ferromagnet Fe_3−x_GaTe_2_ ,” Nature Communications 15, no. 1 (2024): 4472, 10.1038/s41467-024-48799-9. PMC11127993 38796498

[30] S. Rohart and A. Thiaville , “Skyrmion Confinement in Ultrathin Film Nanostructures in the Presence of Dzyaloshinskii‐Moriya Interaction,” Physical Review B 88, no. 18 (2013): 184422, 10.1103/PhysRevB.88.184422.

[31] A. Erickson , Q. Zhang , H. Vakili , et al., “Effect of Magnetic Anisotropy and Gradient‐Induced Dzyaloshinskii‐Moriya Interaction on the Formation of Magnetic Skyrmions,” Small 21, no. 37 (2025): 05204, 10.1002/smll.202505204. PMC12444826 40714808

[32] Y. Ji , S. Yang , H.‐B. Ahn , et al., “Direct Observation of Room‐Temperature Magnetic Skyrmion Motion Driven by Ultra‐Low Current Density in Van Der Waals Ferromagnets,” Advanced Materials 36, no. 21 (2024): 2312013, 10.1002/adma.202312013. 38270245

[33] E. Díaz , A. Anadón , P. Olleros‐Rodríguez , et al., “Energy‐efficient Picosecond Spin–orbit Torque Magnetization Switching in Ferro‐ and Ferrimagnetic Films,” Nature Nanotechnology 20 (2025): 36, 10.1038/s41565-024-01788-x. 39327513

[34] J. Fang , J. Hu , X. Chen , et al., “Electrothermal Manipulation of Current‐Induced Phase Transitions in Ferrimagnetic Mn_3_Si_2_Te_6_ ,” Physical Review Letters 134, no. 25 (2025): 256302, 10.1103/ry2d-bgyy. 40742997

[35] W. Jiang , P. Upadhyaya , W. Zhang , et al., “Blowing Magnetic Skyrmion Bubbles,” Science 349, no. 6245 (2015): 283–286, 10.1126/science.aaa1442. 26067256

[36] W. Legrand , D. Maccariello , N. Reyren , et al., “Room‐Temperature Current‐Induced Generation and Motion of Sub‐100 Nm Skyrmions,” Nano Letters 17, no. 4 (2017): 2703–2712, 10.1021/acs.nanolett.7b00649. 28358984

[37] B. K. Hazra , B. Pal , J.‐C. Jeon , et al., “Generation of out‐of‐plane Polarized Spin Current by Spin Swapping,” Nature Communications 14, no. 1 (2023): 4549, 10.1038/s41467-023-39884-6. PMC10382594 37507398

[38] D. Song , W. Wang , S. Zhang , et al., “Steady Motion of 80‐nm‐size Skyrmions in a 100‐nm‐wide Track,” Nature Communications 15, no. 1 (2024): 5614, 10.1038/s41467-024-49976-6. PMC11224351 38965221

[39] X. Zhang , G. P. Zhao , H. Fangohr , et al., “Skyrmion‐skyrmion and skyrmion‐edge Repulsions in skyrmion‐based Racetrack Memory,” Scientific Reports 5, no. 1 (2015): 7643, 10.1038/srep07643. PMC4284505 25560935

[40] D. Foster , C. Kind , P. J. Ackerman , J.‐S. B. Tai , M. R. Dennis , and I. I. Smalyukh , “Two‐dimensional Skyrmion Bags in Liquid Crystals and Ferromagnets,” Nature Physics 15, no. 7 (2019): 655–659, 10.1038/s41567-019-0476-x.

[41] K. Zeissler , S. Finizio , K. Shahbazi , et al., “Discrete Hall Resistivity Contribution From Néel Skyrmions in Multilayer Nanodiscs,” Nature Nanotechnology 13, no. 12 (2018): 1161–1166, 10.1038/s41565-018-0268-y. 30275493

