# Supplementary material for: Programmable Topological Magnetic Textures in Above‐Room‐Temperature 2D Ferromagnet Fe3GaTe2
Source: Adv Sci (Weinh). 2026 Sep 21:e77160. Online ahead of print. doi: 10.1002/advs.77160 (PMC13591342; doi:10.1002/advs.77160)
Supplement: Supplementary file 1 [file ADVS-9999-e77160-s002.docx]

Programmable Topological Magnetic Textures in Above-Room-Temperature 2D Ferromagnet Fe_3_GaTe_2_

*Hanqing Shi^1^, Yilian Xi^2^, Jingwei Zhang^1^, Jiaqi Liu^1^, Zhizhen Ren^1^, Shilei Zhang^3^, Weichang Hao^1^, Haifeng Feng^1*^, and Yi Du^1*^*

1 School of Physics, Beihang University, Haidian District, Beijing 100191, P. R. China.
2 College of Mathematics and Physics, Beijing University of Chemical Technology, Beijing, 100029, P. R. China.
3 School of Physical Science and Technology, ShanghaiTech University, Pudong New Area, Shanghai 201210, P. R. China.

*Hanqing Shi, Yilian Xi, and Jingwei Zhang* contributed equally to this work.

^*^ Corresponding authors. E-mail: <yi_du@buaa.edu.cn>; [haifengfeng@buaa.edu.cn](mailto:haifengfeng@buaa.edu.cn)

**Contents**

Supplementary Note 1. Energetic model for surface domain branching and selective formation of skyrmion bags.

Table S1. The standard ASCII codes and the corresponding skyrmion bag coding schemes for different letters.

Figure S1. Magnetic domains of FGaT under different magnetic fields.

Figure S2. Determination of the thickness of the sample.

Figure S3. Branched domains in FGaT.

Figure S4. Model of branching domain.

Figure S5. Determination of the critical thickness.

Figure S6. Magnetic domain widths on the sample surface.

Figure S7. Magnetic skyrmions in FGaT.

Figure S8. LTEM images of skyrmion in FGaT.

Figure S9. Reconstruction of the demagnetization field and internal magnetic domain configuration via MFM.

Figure S10. MFM images of skyrmion bags, along with the corresponding phase profile along the white line indicated in the figure.

Figure S11. MOKE images of the magnetic domains in the FGaT sample before and after exfoliation.

Figure S12. Stability of skyrmion bags.

Figure S13. Generation of magnetic skyrmions by field cooling.

Figure S14. Energy diagram during the application of pulse current.

Figure S15. Precise generation of skyrmion bags through current injection.

Figure S16. Device fabrication and current-injection configuration.

Figure S17. Movement of higher-order skyrmion bags.

Figure S18. Movement of lower-order skyrmion bags.

Figure S19. Moving speed under pulse with different current densities.

Figure S20. Relative sliding of skyrmion between high-order skyrmion bags.

**Supplementary Note 1. Energetic model for surface domain branching and selective formation of skyrmion bags**

To provide a quantitative understanding of the proposed “surface–interior energy landscape decoupling” mechanism, we employ an analytical model based on the energetics of surface domain branching in thick magnetic samples.

Following the classical two-phase branching model proposed by Hubert & Schäfer (as illustrated in Figure S4), a sample with a thickness of $D$ is considered. The magnetic domain width in the bulk region is denoted by ($W_{b}$), while the narrower domain width near the surface (where gradual splitting occurs) is denoted by $W_{s}$.

For a 180° Bloch wall, which is the most predominant type in strongly anisotropic magnetic materials, the domain-wall energy density is given by:

$$\gamma_{w}=4\sqrt{AK_{u}}$$

where $A$ is the exchange stiffness and $K_{u}$ is the uniaxial anisotropy constant. Within the Kittel stripe-domain framework, the domain-wall energy density is inversely proportional to the domain width.

$$E_{\text{wall}}=\frac{2\gamma_{w}D}{WD}=\frac{2\gamma_{w}}{W}$$

Narrower domains lead to a higher density of domain walls and consequently an increased domain-wall energy.

If the domain width continuously varies along the sample thickness, the local domain-wall energy can be expressed as:

$$E_{\text{wall}}=2 \int_{0}^{\frac{D}{2}} \left[ \left( \frac{\gamma_{w}}{W\left( z \right)} \right) \right]dz$$

where $W(z)$ represents the local domain width at depth z.

Next, the demagnetization energy consists of two contributions. The branching process inevitably increases the density of internal magnetic charges, thereby giving rise to an internal stray-field energy:

$$E_{D1}= 2 \int_{0}^{\frac{D}{2}} \left[ F_{i}\left( \frac{dW\left( z \right)}{dz} \right)^{2} \right]dz$$

Meanwhile, the external stray-field energy is determined by the domain width at the sample surface:

$$E_{D2}=2C_{S}W_{S}$$

where coefficients $F_{i}$ and $C_{S}$ are phenomenological parameters.

Therefore, the total energy of the system can be expressed as:

$$E_{\mathrm{tot}}= 2 \int_{0}^{\frac{D}{2}} \left[ \left( \frac{\gamma_{w}}{W\left( z \right)} \right)+ F_{i}\left( \frac{dW\left( z \right)}{dz} \right)^{2} \right]dz+ 2C_{S}W_{S}$$

At this stage, the DMI energy is not included in the model. This is justified because the formation of branched domains is primarily governed by the competition between domain-wall energy and magnetostatic energy, whereas the DMI mainly influences the detailed spin configuration and the subsequent stabilization of topological spin textures. When DMI is included, the energy of chiral domain walls is reduced according to $\gamma_{w}=4\sqrt{AK_{u}}-\pi D$. As a consequence, magnetic configurations containing closed chiral domain-wall loops become energetically favorable. Therefore, DMI primarily influences the chirality of magnetic domain walls, but does not directly determine the occurrence of surface domain branching.

Minimizing the total-energy expression produces a critical thickness, below which the uniform stripe-domain state is stable, and above which the surface-branched state becomes energetically favorable:

$$D_{s}=\frac{1}{8}\pi^{4}\gamma_{w}F_{i}^{2}/C_{s}^{3}$$

The model further predicts that, in the large-thickness limit, the surface domain width approaches a saturated value:

$$W_{s}=4\gamma_{w}F_{i}/C_{s}^{2}$$

Experimental observations are consistent with these predictions. As shown in Figure S5, a clear critical thickness is observed in a gradient-thickness sample. Surface domain branching, characterized by the coexistence of skyrmions and skyrmion bags, appears only in the thick region of the sample ($10 \mu m$), whereas no branching is observed in thinner regions ($5-7 \mu m$). Thus, the experimental critical thickness is determined to be $D_{s}\approx10 \mu m$. Furthermore, the measured widths of the bifurcated surface domains stabilize within a range of $W_{S}\approx0.5 \mu m\sim2 \mu m$ (Figure S6).

The energetic model also provides a framework for understanding the selective formation of skyrmion bags under current pulses. In the bulk region, where domain branching is absent, the dominant energy contribution originates from domain walls:

$${E_{total}=E}_{\text{wall}}=\frac{2\gamma_{w}}{W_{1}}$$

In contrast, the surface-branched region contains both domain-wall and magnetostatic contributions:

$${E_{total}=E}_{\text{wall}}+E_{D1}=\frac{2\gamma_{w}}{W_{2}}+E_{D1}$$

Since domain branching leads to narrower surface domains ($W_{2}<W_{1}$), the density of domain walls increases, resulting in a larger domain-wall energy density in the surface region. Furthermore, the presence of the additional stray-field energy $E_{D1}$further increases the total energy density.

Therefore,

$$E_{surface}>E_{bulk}$$

This surface–bulk energetic differentiation provides a physical basis for the selective manipulation of surface magnetic domains by electrical current. When a current is applied, Joule heating introduces an additional energy input into the system ($E_{current}$). This energy input enables the system to overcome local pinning barriers ($E_{\text{barrier}}$) in the free-energy landscape. Crucially, there exists an intermediate current regime where the combined energy satisfies:

$$E_{\text{surface}}+E_{\text{current}}>E_{\text{barrier}},$$

While:

$$E_{\text{bulk}}+E_{\text{current}}<E_{\text{barrier}}.$$

Under this condition, the energy input is sufficient to trigger a reconstruction of the unstable surface domains, while the interior bulk configuration remains well-preserved.

Since the bulk magnetic structure is preserved, it acts as a magnetic framework that constrains the evolution of the surface spin texture. As a result, the reconstructed surface domains naturally evolve into skyrmion-bag configurations that are consistent with the underlying bulk-domain architecture.

Therefore, the selective formation of skyrmion bags is a direct consequence of the energetic differentiation between the surface and bulk regions, combined with the carefully controlled current-induced energy input.

| **Alphabet** | **ASCII code** | **Coding** |
| --- | --- | --- |
| A | 65 | 01000001 |
| B | 66 | 01000010 |
| C | 67 | 01000011 |
| D | 68 | 01000100 |
| E | 69 | 01000101 |
| … | … | … |
| Z | 90 | 01011010 |

**Table S1. The standard ASCII codes and the corresponding skyrmion bag coding schemes for different letters.**

**Figure S1. Magnetic domains of FGaT under different magnetic fields.** The domains of FGaT with a thickness of 3 μm were obtained by magneto-optical Kerr microscope. The inset shows the three-dimensional structure of labyrinthine domain of FGaT. Scale bar: 5 μm.

**Figure S2.** **Determination of the thickness of the sample.** Optical microscope image of the cross-section of the sample used for testing.

**Figure S3. Branched domains in FGaT.**

**Figure S4. Model of branching domain.**

**Figure S5.** **Determination of the critical thickness.** (a) A sample with a gradient thickness profile was prepared, with thicknesses of 10 μm, 7 μm, and 5 μm at the thick end, middle region, and thin end, respectively. (b) Thickness variation diagram of the sample. (c) MOKE image of the sample. At the thick end, the magnetic domains exhibit incipient surface splitting, evidenced by the coexistence of isolated skyrmions and skyrmion bags. In contrast, only skyrmions were observed in the middle and thin regions, confirming that surface domain splitting does not occur below this critical thickness.

**Figure S6. Magnetic domain widths on the sample surface.** (a) Optical microscope image of the sample. The thickness of the sample is determined to be 176 μm. (b) Magnetic force microscopy (MFM) image of this sample. The surface magnetic domains exhibit progressive splitting, with the widths of these bifurcated domains gradually stabilizing within a well-defined range.

**Figure S7. Magnetic skyrmions in FGaT.** FGaT of different thicknesses were obtained via tape exfoliation, and skyrmions were generated through field cooling. The magnetic domain information was characterized by MFM.

**Figure S8. LTEM images of skyrmion in FGaT.** LTEM measurement results of FGaT flakes at two different thicknesses (0.1 μm and 0.2 μm) under different tilt angles (0°, +20°, and −20°). Scale bar: 500 nm.

**Figure S9. Reconstruction of the demagnetization field and internal magnetic domain configuration via MFM.** MFM measurements were performed at lift heights of 100 nm, 500 nm, 1000 nm, and 2000 nm, respectively. Since MFM probes the stray field intensity at the sample surface, the surface demagnetization field distribution can be reconstructed, which in turn enables the recovery of the internal magnetic domain structure within the sample.

**Figure S10. MFM images of skyrmion bags, along with the corresponding phase profile along the white line indicated in the figure.**

**Figure S11. MOKE images of the magnetic domains in the FGaT sample before and after exfoliation.** (a) Magnetic domains before exfoliation. (b) Magnetic domains after exfoliation. Note that the domain images acquired before and after exfoliation correspond to the same sample region, and each individual domain can be matched one-to-one between the two images. The black spots visible in the images are dead pixels originating from the camera.

**Figure S12. Stability of skyrmion bags.** (a) MOKE images of skyrmion bags induced in a 20-μm-thick FGaT. (b) MFM image of the skyrmion bags in the same sample after 1 year of exposure to air.

**Figure S13. Generation of magnetic skyrmions by field cooling.** The labyrinthine domains undergo transformation into skyrmions in FGaT with a thickness of 102 nm.

**Figure S14. Energy diagram during the application of pulse current.** During the process of current application, the system's energy surpasses the energy barrier, thereby inducing a transition from labyrinthine domains to skyrmion bags.

**Figure S15. Precise generation of skyrmion bags through current injection.** The magnetic domains can progressively achieve higher-order topological numbers under the applied current (*j* = 8 × 10^9^ A/m², τ = 550 μs).

**Figure S16. Device fabrication and current-injection configuration.** The FGaT sample was fabricated in a strip geometry. A liquid-metal GaInSn alloy was employed as the electrical contact, providing excellent electrical coupling with the FGaT crystal. Owing to the much lower resistance of the liquid metal compared with that of FGaT, the resistance contribution from the external circuit can be neglected during current injection. The two liquid-metal electrodes were arranged in a parallel configuration to ensure a uniform current distribution across the sample.

**Figure S17. Movement of higher-order skyrmion bags (the number of next-nearest-neighbor magnetic domains N^N^** **> 0).**

**Figure S18. Movement of lower-order skyrmion bags (such as the domain with Q = 0).**

**Figure S****19. Moving speed under pulse with different current densities.**

**Figure S20. Relative sliding of skyrmion between high-order skyrmion bags.** After applying 300 current pulses, it is found that skyrmions have relative motion in addition to the overall motion. The yellow dotted line in the figure indicates the trajectory of relative motion.
